# Effects of Culling on *Leptospira interrogans* Carriage by Rats

**DOI:** 10.3201/eid2402.171371

**Published:** 2018-02

**Authors:** Michael J. Lee, Kaylee A. Byers, Christina M. Donovan, Julie J. Bidulka, Craig Stephen, David M. Patrick, Chelsea G. Himsworth

**Affiliations:** The University of British Columbia, Vancouver, British Columbia, Canada (M.J. Lee, K.A. Byers, C.M. Donovan, D.M. Patrick, C.G. Himsworth);; Canadian Wildlife Health Cooperative, Abbotsford, British Columbia, Canada (M.J. Lee, K.A. Byers, C. Stephen, C.G. Himsworth);; British Columbia Ministry of Agriculture, Abbotsford (J.J. Bidulka, C.G. Himsworth);; University of Saskatchewan, Saskatoon, Saskatchewan, Canada (C. Stephen);; British Columbia Centre for Disease Control, Vancouver (D.M. Patrick)

**Keywords:** Leptospira interrogans, leptospirosis, rodent control, zoonoses, Norway rat, bacteria, Canada, culling, rats, carriage

## Abstract

We found that lethal, urban rat control is associated with a significant increase in the odds that surviving rats carry *Leptospira interrogans*. Our results suggest that human interventions have the potential to affect and even increase the prevalence of zoonotic pathogens within rat populations.

Norway rats (*Rattus norvegicus*) are a reservoir for *Leptospira interrogans*, the etiologic agent of the zoonotic disease leptospirosis (*1*). Leptospirosis affects ≈1 million persons worldwide annually and can result in kidney failure or pulmonary hemorrhage (*1**,**2*). Increasing urbanization has driven the emergence of leptospirosis in cities globally (*3*). Within cities, areas of poverty experience a confluence of environmental and socioeconomic factors that heighten the risk for ratborne *L. interrogans* transmission (*3*).

The ecology of rats and the epidemiology of *L. interrogans* within their populations are intimately connected (*4*). Previous research on other reservoir species suggests that anthropogenic disturbances may alter reservoir ecology, resulting in new transmission patterns (*5**,**6*). Because lethal control is a common technique used to address rat populations (*7**,**8*), we aimed to determine whether culling effects *L. interrogans* carriage by urban Norway rats.

## The Study

**Figure 1:**
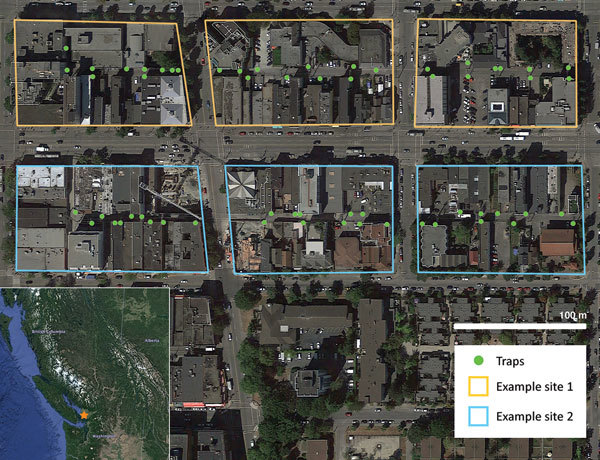
Two example sites side-by-side in a study of the effects of culling on *Leptospira interrogans* carriage by rats, Vancouver, British Columbia, Canada, June 2016–January 2017. Each site comprised 3 city blocks connected by continuous alleys; individual sites that were trapped at the same time had parallel alleys separated by major roads and multiple buildings that, based on previous research (*9**,**10*), rats were assumed to be unlikely to move between. Five and 7 sites were randomly selected as intervention and control sites, respectively. In intervention sites, kill-trapping was conducted in the center of the 3 blocks; blocks flanking the intervention block were designated nonkill flanking blocks (nonkill flanking blocks were trapped to detect any indirect effects of kill-trapping, such as immigration from/emigration to the intervention block). Image downloaded from Google Earth Professional (https://www.google.com/earth/download/gep/agree.html).

**Figure 2:**
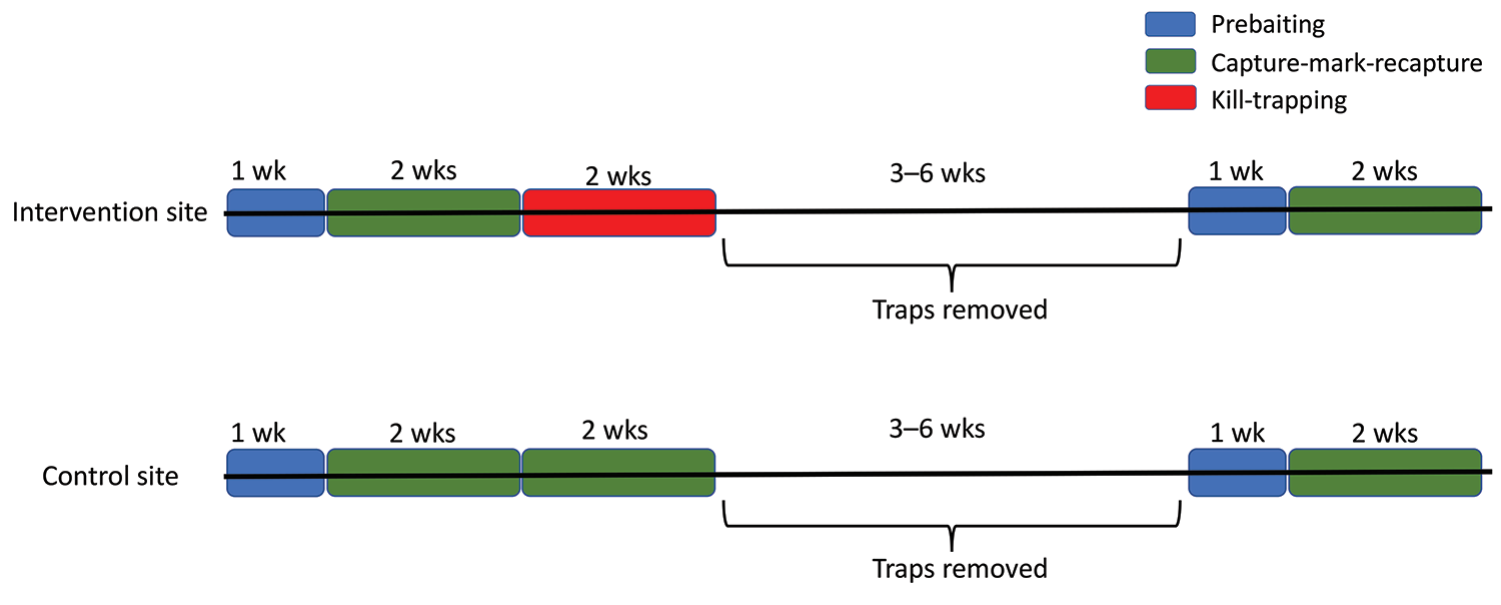
Experiment timeline in intervention and control sites in a study of the effects of culling on *Leptospira interrogans* carriage by rats, Vancouver, British Columbia, Canada, June 2016–January 2017. Trapping in each intervention site was divided into three 2-week periods: the period before kill-trapping, the period during kill-trapping, and the period after kill-trapping. During the 2 weeks before kill-trapping, we captured and sampled rats, gave them all a unique ear-tag identifier, and then released them where they were caught. In the following 2 weeks (the kill-trapping period) rats that were caught in the center of the 3 blocks were euthanized; catch-release continued in flanking blocks. Traps were then removed for >3–6 weeks, after which they were returned to their exact prior locations, and capture-sample-release continued for 2 more weeks (the period after kill-trapping). The trapping protocol was the same for control blocks except that capture-sample-release was conducted during all 2-week trapping periods. Prebaiting (during which traps were fixed open) was used to acclimate rats to cages (Technical Appendix).

This study, conducted in an inner-city neighborhood of Vancouver, British Columbia, Canada, during June 2016–January 2017, compared the prevalence of *L. interrogans* in rat populations before and after a kill-trapping intervention. Each study site (12 total) comprised 3 contiguous city blocks and was designated as a control site or an intervention site (Figure 1). In control sites, no kill-trapping occurred; in intervention sites, kill-trapping occurred only in the central blocks, and the 2 adjacent blocks were designated as nonkill flanking blocks. We divided trapping in each intervention site into 3 time periods: before, during, and after the intervention (Figure 2). Before and after the intervention, rats were trapped, processed, and released. During processing, rats were marked with an ear tag, and morphometric information was recorded (Table 1). Urine was obtained from these rats and tested for *L. interrogans* by real-time PCR. During the intervention, we euthanized trapped rats; in control sites and flanking blocks, capture-release continued, and rats were not euthanized. The University of British Columbia’s Animal Care Committee (A14-0265) approved all procedures (Technical Appendix).

**Table 1:** Distributions of covariates by rat-trapping period and *Leptospira interrogans* real-time PCR status, Vancouver, British Columbia, Canada, June 2016–January 2017*

Covariate	Total	PCR status before intervention			PCR status after intervention	
		Negative	Positive		Negative	Positive
Total	430	226	39		140	25
Season, no. (%)						
Summer, Jun–Aug	115 (27)	83 (37)	15 (38)		13 (9)	4 (16)
Fall, Sep–Nov	203 (47)	143 (63)	24 (62)		33 (24)	3 (12)
Winter, Dec–Feb	112 (26)	0†	0†		94 (67)	18 (72)
Sex, no. (%)						
F	205 (48)	107 (47)	16 (41)		69 (49)	13 (52)
M	225 (52)	119 (53)	23 (59)		71 (51)	12 (48)
Sexual maturity, no. (%)						
Juvenile	178 (41)	117 (52)	1 (3)		56 (40)	4 (16)
Mature	252 (59)	109 (48)	38 (97)		84 (60)	21 (84)
Continuous median length, cm (IQR)	31 (26–39)	29 (25–37)	41 (36–43)		30 (26–36)	39 (33–42)
Wounds, no. (%)						
No	316 (73)	173 (77)	11 (28)		115 (82)	17 (68)
Yes	114 (27)	53 (23)	28 (72)		25 (18)	8 (32)
Weight, g, no. (%)						
<122	212 (49)	129 (57)	1 (3)		77 (55)	5 (20)
>122	218 (51)	97 (43)	38 (97)		63 (45)	20 (80)

*IQR, interquartile range. †No periods before the intervention period were conducted during winter.

We used mixed-effects multivariable logistic regression to estimate the effect of the intervention on the odds that rats carried *L. interrogans*, while controlling for clustering by city block (*4*). The outcome was the *L. interrogans* PCR status (negative or positive) of individual rats. The predictor variable categorized rats by block and period of capture: 0, rats caught before the intervention; 1, rats caught after the intervention in control blocks; 2, rats caught after the intervention in nonkill flanking blocks; and 3, rats caught after the intervention in intervention blocks. Although we did not undertake the intervention in control sites, we considered the third 2-week trapping period independently from the other trapping periods in control sites to detect any temporal changes in *L. interrogans* prevalence not associated with the intervention. We excluded the 7 rats captured both before and after the intervention to avoid double-counting individual rats. For rats recaptured within the same period as their first capture (either before or after the intervention), we averaged weight and length across captures. We also excluded 1 rat missing data for covariates under consideration.

We used a hypothesis-testing model-building approach to estimate the effect of the intervention while controlling for covariates (Table 1). We kept covariates, selected on the basis of their potential to confound the relationship between the intervention and *L. interrogans* carriage, in the model if they changed the estimated relationship between the predictor and outcome variables by >10%. Because length and weight were collinear, we used the covariate with the largest effect on the relationship between the predictor and outcome. We dichotomized weight around its median because it was not linear with the log-odds of the outcome. For statistical analyses, we used RStudio (Boston, MA, USA).

Of the 438 rats trapped, we included 430 in the modeling process (Table 1). Sixty-four (14.9%; 95% CI 11.7%–18.7%) rats were PCR-positive for *L. interrogans*. Of 131 rats recaptured, 5 were *L. interrogans* positive at their first capture and recapture; no positive rats changed pathogen status within a trapping period.

Rats caught in intervention blocks after an intervention had 9.55 times the odds of carrying *L. interrogans* than did rats trapped before an intervention, while adjusting for weight and wound presence variables (Table 2). We found no significant changes in either flanking blocks or control blocks. In this model, 52.6% of the total model variance was due to the random effect of the block (*11*). Rerunning the final model including animals that were caught both before and after the intervention did not substantially affect the results (effect of the intervention in intervention blocks; adjusted odds ratio 8.88, 95% CI 1.68–68.08).

**Table 2:** Results of model building in a study of the effects of culling on *Leptospira interrogans* carriage by rats, Vancouver, British Columbia, Canada, June 2016–January 2017

Covariate	Unadjusted odds ratio* (95% CI)	Adjusted odds ratio† (95% CI)	p value
Season			
Summer	Reference	–‡	–
Fall	0.44 (0.13–1.39)	–	–
Winter	0.87 (0.22–3.24)	–	–
Sex			
F	Reference	–	–
M	1.28 (0.70–2.37)	–	–
Sexual maturity			
Juvenile	Reference	–	–
Mature	16.26 (6.28–51.95)	–	–
Continuous length, cm	1.25 (1.18–1.35)	–	–
Wounds			
No	Reference	Reference	
Yes	1.81 (1.42–2.39)	3.87 (1.73–9.12)	0.0013
Weight, g			
<122	Reference	Reference	
>122	17.88 (7.22–53.28)	9.98 (3.70–31.74)	<10^–4^
Intervention			
Before intervention, all block types, n = 261	Reference	Reference	
After intervention, control blocks, n = 97	0.69 (0.22–2.00)	0.77 (0.22–2.58)	0.68
After intervention, nonkill flanking blocks, n = 33	1.50 (0.49–4.40)	2.22 (0.65–7.47)	0.19
After intervention, intervention blocks, n = 39	8.67 (2.02–55.00)	9.55 (1.75–78.31)	0.016

*Bivariable relationships between the indicated covariate and *L. interrogans* status, while controlling for the random effect of the block. †Results of the final multivariable model in which the effect of each covariate is adjusted for other covariates in the model. ‡Dashes indicate variables not carried forward into the final multivariable model on the basis of statistical confounding criteria.

## Conclusions

This study showed that kill-trapping was associated with increased odds that rats carried *L. interrogans* in the city blocks where kill-trapping occurred. We did not observe this effect in control blocks or nonkill flanking blocks.

Increased intraspecific transmission of *L. interrogans* resulting from kill-trapping is a plausible explanation for the observed effect. Previous research suggests that rat-to-rat transmission of *L. interrogans* is associated with social structures in rat colonies (*4*). Given that culling is ineffective at removing entire rat populations (*7**,**8**,**12*), kill-trapping may have disrupted social structures and promoted new interactions that facilitated transmission among remaining rats. For example, culling may have removed dominant rats (*13*), subsequently increasing aggressive interactions among the remaining rats as they established a new social hierarchy. The positive association between *L. interrogans* status and weight/wound presence (which are correlated with hierarchical dominance) supports this hypothesis because the bacteria may be transmitted through specific aggressive/dominance interactions (*4*).

We assessed only the effect of culling on a single ratborne pathogen. *L. interrogans* might be particularly susceptible to the effects of culling because of its dependence on rat social structures. Other vectorborne (e.g., fleaborne *Rickettsia* spp. [*14*]) or environmentally acquired (e.g., methicillin-resistant *Staphylococcus aureus* [*15*]) rat-associated pathogens might not be as easily influenced by culling. Future studies should determine the duration of effects induced by lethal control because effects on *L. interrogans* prevalence may wane with time. However, given that such methods are ineffective at removing entire rat populations and might therefore be used repeatedly as the population rebounds (*7**,**8**,**12*), short-term effects may be particularly important.

We demonstrated that rat culling has the potential to increase the odds for *L. interrogans* carriage among remaining rats and thus could potentially increase the risk for transmission to humans. Although public health risks resulting from such an increase postintervention might be offset by a decrease in the number of rats, we were unable to quantify the size of the rat population before and after intervention. Practical and ethical considerations make it difficult to empirically demonstrate a direct link between culling and increased pathogen transmission from rats to humans. Rather, after culling, the potential for a person to encounter a rat carrying *L. interrogans* increases if a person encounters a rat, suggesting that the risk for zoonotic transmission increases per rat contact.

The convergence of this study with previous literature documenting that reactive culling is often unsuccessful at removing rat populations (*7**,**8**,**12*) indicates that such methods are ineffective. Instead, ecologically based rodent management, which focuses on reducing resources available to rats (*8*), should be more widely applied to urban environments.

By integrating our results with other studies on the impacts of culling wild animals to control communicable diseases (*5**,**6*), we can conclude that killing animal reservoirs of human pathogens might have unintended consequences on the disease risks. This hypothesis underscores the importance of understanding the ecology of the targeted animal reservoir to design effective control programs.

Technical Appendix Additional methods for a study of the effects of culling on *Leptospira interrogans* carriage by rats, Vancouver, British Columbia, Canada, June 2016–January 2017.
