# Supplementary material for: Effects of Culling on Leptospira interrogans Carriage by Rats
Source: Emerg Infect Dis. 2018 Feb;24(2):356–60. doi: 10.3201/eid2402.171371 (PMC5782904; doi:10.3201/eid2402.171371)
Supplement: Technical Appendix — Additional methods for a study of the effects of culling on Leptospira interrogans carriage by rats, Vancouver, British Columbia, Canada, June 2016–January 2017. [file 17-1371-Techapp-s1.pdf]

Article DOI: http://dx.doi.org/10.3201/eid2402.171371

Effects of Culling on *Leptospira interrogans* Carriage by Rats

Technical Appendix

Real-Time PCR

Nucleic acid from rat urine was extracted with a 96-well magnetic particle processor using the MagMAX Pathogen RNA/DNA kit (Life Technologies). Extractions were performed following the procedure outlined by the manufacturer for low-cell content samples. Starting urine volume ranges were 20–200 μL, and all were volume corrected to 200 μL using sterile, 1× phosphate buffer solution buffer, pH 7.4.

Nucleic acid extracts were amplified using a real-time PCR (Life Technologies) that targets the LipL32 gene (encodes an outer membrane lipoprotein virulence factor [*1*] of pathogenic *Leptospira* spp.). Real-time PCR was performed using the Agpath-ID One-Step real-time PCR Kit (Life Technologies). A Taqman exogenous internal positive control (IPC) (Life Technologies) was also run to ensure that there was no PCR inhibition due to the inhibitory nature of urine samples.

Each 25-µL reaction contained 2× real-time PCR buffer, 25X real-time PCR enzyme, 800 nM each of forward primer (5′-AAG CAT TAC CGC TTG TGG TG-3′) and reverse primer (5′-GAA CTC CCA TTT CAG CGA TT-3′), 200-nM probe (5′-FAM/AAA GCC AGG ACA AGC GCC G/BHQ1-3′), 10X Exo IPC Mix, 500× Exo IPC DNA (diluted 10-fold), nuclease-free water and 5 µL of DNA template. The reaction was incubated at 50°C for 2 min, 95°C for 10 min, and then amplified for 45 cycles at 95°C for 15 s, 58°C for 1 min. Samples were run on an ABI7500 Fast PCR system (Life Technologies) and analyzed using the SDS software version 1.4 (Life Technologies).

Leptospira spp. primers and probe were made by Integrated DNA Technologies (San Diego, CA, USA). A negative extraction control, negative template control, and 2 positive

amplification controls were used per real-time PCR run. The positive control was *L. interrogans*, serovar *copenhageni* (Tim Witchell, University of Victoria, BC, Canada, June 2012).

Field Methods

Trapping

Trapping was conducted during June 2016–January 2017. Ten Tomahawk Rigid Traps (Tomahawk Live Traps, Hazelhurst, WI, USA) were placed in the alley that bisected each city block. To prevent vandalism, traps were fitted into stainless steel trap covers (Integrated Pest Supplies Ltd, New Westminster, BC, Canada) and chained to immovable objects. Traps were baited with peanut butter mixed with oats. Hydrogel (ClearH2O, Westbrook, ME, USA) was provided as a water source.

Three study sites were trapped at a time, such that 90 traps were deployed in 9 city blocks at any given point during this study. Prebaiting, in which cages were fixed open and baited, was conducted for 1 week before any new trapping period to acclimatize rats to cages. During trapping periods, traps were set each evening by 4 PM and checked each morning by 7 AM, 5 days a week. On the sixth and seventh days, traps were fixed open and baited. Traps and associated equipment were sanitized in 10% bleach and/or 70% ethanol (*2*) after coming into contact with any rat and after any period of prebaiting.

Sample Collection

Captured rats were transported to the back of a mobile laboratory van and given Hydrogel to promote urination, and their cages were covered with a blanket to minimize stress until sampling. Urine was obtained by placing caged rats directly above a bleach-sanitized plastic tray until they urinated into it. Urine was collected using a sterile syringe and was stored at –80°C until analysis. Subsequently, rats were transferred into an inhalation induction chamber (Kent Scientific, Torrington, CT, USA) and anesthetized with 5% isoflurane in oxygen using an isoflurane vaporizer (Associated Respiratory Veterinary Services, Lacombe, AB, Canada). Anesthesia was maintained throughout sampling.

Each rat was given a unique laser-etched ear-tag (Kent Scientific) for identification upon recapture. The following demographic and morphometric characteristics were assessed: body weight (grams), total length (nose-to-tail in centimeters), sexual maturity (males with scrotal

testes and females with a perforate vagina were considered mature), sex (male or female), and the presence/absence of bite wounds (presence determined in accordance with [*3*]). Rats were allowed to recover fully from anesthesia before being released at the exact location of their capture ≈15–30 minutes after sampling.

Rats that had been previously captured and sampled were resampled if >7 days had passed since their previous capture. One week was determined to be an appropriate interval in which to detect a change in *L. interrogans* infection status because the bacterium can be detected in renal tissue and rat urine in as little as 1 week after experimental infection (*4,5*). Rats caught in intervention blocks during the 2-week kill-trapping period were anesthetized using isoflurane and euthanized by intracardiac injection with pentobarbital.

Interactions

We explored biologically plausible interactions between the effect of the intervention and covariates by running the final multivariable model in strata of each covariate. For example, we hypothesized that effect of the intervention on *L. interrogans* carriage might be modified by socially relevant morphologic characteristics, such as sexual maturity, given that transmission between rats might depend on social structures (*6*). For sexual maturity, we therefore stratified and recomputed the multivariable model for juvenile and mature rats separately. However, because of a limited sample size, there was not enough statistical power to test for interactions in the restricted strata.

Of all covariates tested, sex was the only variable that had large enough strata to test for interactions. These data indicated that the effect of the intervention may be more pronounced among females (adjusted odds ratio 7.62, 95% CI 0.81–110.49) than among males (adjusted odds ratio 3.71, 95% CI 0.38–63.45), while weight and bite wounds remained constant. However, the effect of the intervention was not significant in either strata, again, most likely because of the limited sample size in each group.

References

1. Stoddard RA, Gee JE, Wilkins PP, McCaustland K, Hoffmaster AR. Detection of pathogenic *Leptospira* spp. through TaqMan polymerase chain reaction targeting the LipL32 gene. Diagn

Microbiol Infect Dis. 2009;64:247–55. PubMed http://dx.doi.org/10.1016/j.diagmicrobio.2009.03.014

2. Health Canada, Public Health Agency of Canada. Pathogen safety data sheets: infectious substances—*Leptospira interrogans*. p. 1–3. [cited 2017 Jun 8]. http://www.phac-aspc.gc.ca/lab-bio/res/psds-ftss/msds95e-eng.php

3. Himsworth CG, Zabek E, Tang P, Parsons KL, Koehn M, Jardine CM, et al. Bacteria isolated from conspecific bite wounds in Norway and black rats: implications for rat bite–associated infections in people. Vector Borne Zoonotic Dis. 2014;14:94–100. PubMed http://dx.doi.org/10.1089/vbz.2013.1417

4. Monahan AM, Callanan JJ, Nally JE. Proteomic analysis of *Leptospira interrogans* shed in urine of chronically infected hosts. Infect Immun. 2008;76:4952–8. PubMed http://dx.doi.org/10.1128/IAI.00511-08

5. Athanazio DA, Silva EF, Santos CS, Rocha GM, Vannier-Santos MA, McBride AJA, et al. *Rattus norvegicus* as a model for persistent renal colonization by pathogenic *Leptospira interrogans*. Acta Trop. 2008;105:176–80. PubMed http://dx.doi.org/10.1016/j.actatropica.2007.10.012

6. Himsworth CG, Bidulka J, Parsons KL, Feng AYT, Tang P, Jardine CM, et al. Ecology of *Leptospira interrogans* in Norway rats (*Rattus norvegicus*) in an inner-city neighborhood of Vancouver, Canada. PLoS Negl Trop Dis. 2013;7:e2270–9. PubMed http://dx.doi.org/10.1371/journal.pntd.0002270
